# Interferon signaling in myocardial infarction: mechanistic insights and translational prospects

**DOI:** 10.3389/fimmu.2026.1914545

**Published:** 2026-10-09

**Authors:** Shihan Xu, Yanfei Liu, Mengmeng Zhu, Fengqin Xu, Yue Liu

**Affiliations:** 1 National Clinical Research Center for TCM Cardiology, Xiyuan Hospital of China Academy of Chinese Medical Sciences, Beijing, China; 2 The Second Department of Geriatrics, Xiyuan Hospital of China Academy of Chinese Medical Sciences, Beijing, China; 3 Key Laboratory of Disease and Syndrome Integration Prevention and Treatment of Vascular Aging, Xiyuan Hospital of China Academy of Chinese Medical Sciences, Beijing, China

**Keywords:** cGAS, inflammation, interferon, myocardial infarction, STING

## Abstract

Inflammation following myocardial infarction (MI) is a key determinant of patient prognosis; however, existing anti-inflammatory strategies remain limited in terms of overall benefit, patient eligibility, and safety, creating an urgent need for the development of new anti-inflammatory approaches. Interferon (IFN) signaling permeates the entire process of inflammation and repair following MI. During the inflammatory phase, the cGAS-STING-mediated IFN-I response promotes inflammatory damage; during the proliferative phase, sustained IFN signaling delays the resolution of inflammation and impedes scar formation, whereas moderate IFN signaling promotes resolution; during the maturation phase, IFN signaling can both limit excessive fibrosis and promote low-grade inflammation, scar instability, and the progression of ischemic heart failure. Therefore, precisely identifying the spatiotemporal windows with clear pathogenic roles is of great significance for the translation of IFN signaling. Compared to the bidirectional effects of IFN signaling during the proliferation and maturation phases, the cGAS-STING axis, with its clearly pro-inflammatory role during the inflammatory phase, holds greater translational potential. The development of tissue-specific cGAS-STING inhibitors based on nanodelivery, restricted to short-term application during the acute inflammatory phase of MI, may offer a new therapeutic direction for post-MI anti-inflammatory strategies. Furthermore, in-depth analysis of IFN signaling in conjunction with clinical cohorts in future studies will further aid in the development of precise immunomodulatory strategies for MI.

## 1 Introduction

Myocardial infarction (MI) remains an important cause of cardiovascular mortality and ischemic heart failure worldwide (1). Early reperfusion and guideline-directed medical therapy have reduced mortality after MI, but many patients still develop adverse outcomes, including ventricular dilation and impaired systolic function (2). Inflammation after MI has an important role in determining subsequent repair and remodeling. A controlled inflammatory response helps remove necrotic tissue and supports reparative fibrosis and scar formation (3). In contrast, excessive or prolonged inflammation may worsen cardiomyocyte injury, promote adverse ventricular remodeling, and contribute to progressive cardiac dysfunction (4). Clinical studies have shown that inflammation can be targeted after MI, although the benefits have been variable and safety concerns remain. In CANTOS, inhibition of interleukin-1β (IL-1β) reduced recurrent cardiovascular events in patients with prior MI and elevated high-sensitivity C-reactive protein, but this benefit was accompanied by an increased risk of infection (5). In COLCOT, low-dose colchicine reduced ischemic events when given after MI (6). However, CLEAR SYNERGY (OASIS 9) found that long-term low-dose colchicine, initiated within 72 hours after first percutaneous coronary intervention (PCI) for acute MI, did not significantly reduce major adverse cardiovascular events (7). The VCUART studies suggested that the recombinant IL-1 receptor antagonist anakinra can reduce inflammatory activity after acute MI and may lower heart failure–related events, but its long-term clinical benefit and the patients most likely to benefit require further study (8). Together, these findings support inflammation as a modifiable process after MI, while showing that efficacy depends on the target, timing, disease stage, and treatment-related risks, particularly infection. This variability highlights the need to move from broad-spectrum anti-inflammatory therapy toward mechanistically precise and safer immunomodulation based on a deeper understanding of the post-MI inflammatory network.

Interferon (IFN) signaling represents an underestimated but promising regulatory axis in post-MI inflammation. IFNs were first described for their antiviral activity and are now known to regulate immune homeostasis, cell proliferation, and differentiation (9, 10). Experimental studies suggest that ischemic cardiomyocyte necrosis can activate interferon regulatory factor 3 (IRF3) and type I interferon (IFN-I) responses (11). This activation increases post-MI inflammation and contributes to adverse ventricular remodeling. In these models, inhibition of IRF3 or blockade of IFN receptors reduces inflammation, improves left ventricular function, and increases survival. Nonetheless, the biological functions of post-MI IFN signaling are complex. The effects vary with IFN subtype, cellular source, disease stage, and downstream interferon-stimulated gene (ISG) programs. These factors differentially shape inflammation, fibroblast activation, extracellular matrix deposition, and scar maturation (12). Premature or excessive IFN inhibition may impair essential debris clearance and host defense, whereas nonselective blockade of downstream JAK-STAT pathways can induce systemic immunosuppression (13). These context-dependent effects identify IFN signaling as a promising therapeutic axis but also highlight the need to define its stage- and cell-specific roles. Accordingly, this review systematically examines how IFN signaling initiates and amplifies post-MI inflammation, regulates the transition to repair, and shapes fibrosis and scar maturation. It further evaluates therapeutic opportunities and translational limitations across IFN pathways and their downstream targets to inform precise anti-inflammatory strategies after MI.

## 2 Interferon signaling network

IFNs were initially recognized for their antiviral activity, but their biological effects extend beyond host defense against infection (14). In recent years, IFN signaling has been increasingly studied in the pathophysiology of cardiovascular diseases (12). In MI, IFN signaling can convert tissue injury signals into an antiviral-like transcriptional program. Together with nuclear factor κB (NF-κB) signaling and inflammasome activation, this program contributes to postinfarction inflammation and may influence subsequent repair (15, 16). Because current evidence in MI is mainly focused on IFN-I and type II interferon (IFN-II), a structured review of their biological features, signaling pathways, and interactions with inflammatory networks is needed to clarify their roles in post-MI injury and repair (**Figure 1**).

**Figure 1:**
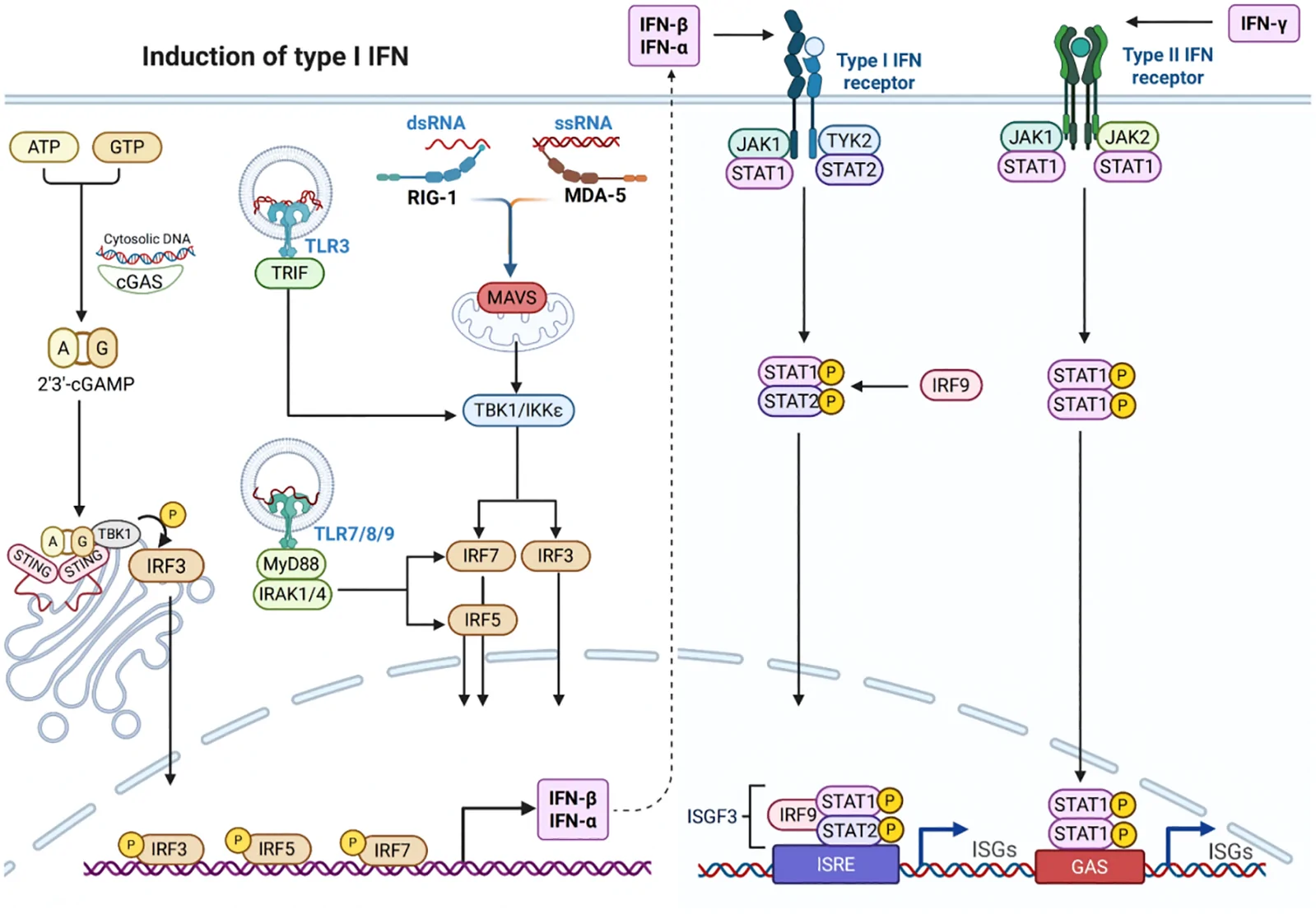
Interferon signaling network. DAMPs can be recognized by the endosomal TLRs 3, 7, 8, and 9; the cytoplasmic RNA receptors RIG-I and MDA5; and the DNA receptor cGAS. TLR3 activates IRF3 and IRF7 via the TRIF-TBK1/IKKϵ axis; TLR7/8/9 primarily activate IRF5/IRF7 via the MyD88-IRAK1/4 pathway; RIG-I and MDA5 recruit TBK1/IKKϵ via MAVS after recognizing abnormal RNA; cytoplasmic DNA is catalyzed by cGAS to generate cGAMP, which activates STING, subsequently recruiting TBK1 and phosphorylating IRF3. These pathways collectively drive the transcription of IFN-I genes, making IFN-I a key hub that converts endogenous damage signals into an immune amplification program following MI. Secreted IFN-I activates JAK1 and TYK2 via IFNAR1/IFNAR2, induces the phosphorylation of STAT1/STAT2, and forms an ISGF3 complex with IRF9, which subsequently binds to ISRE elements to initiate the expression of interferon-stimulated genes (ISGs); IFN-γ, as the sole IFN-II, is primarily derived from activated T cells, NK cells, and NKT cells. It activates JAK1/JAK2 via IFNGR1/IFNGR2, induces the formation of a STAT1 homodimer to form GAF, and binds to GAS elements to regulate ISG transcription.

### 2.1 Interferon signal

IFN belong to the class II α-helical cytokine family and are classified as type I, type II, and type III IFN according to receptor specificity, molecular structure, and evolutionary relationship (17). All three classes can induce antiviral and immunomodulatory programs, but they differ in ligand composition, receptor distribution, cellular sources, and tissue range. These differences help explain their variable effects across pathological settings (18). IFN-I mainly include IFN-α and IFN-β and signal through the IFNAR1/IFNAR2 receptor complex (19, 20). Nearly all nucleated cells can induce IFN-I expression after sensing pathogen-associated molecular patterns (PAMPs) or damage-associated molecular patterns (DAMPs), and important cellular sources include plasmacytoid dendritic cells, monocytes/macrophages, fibroblasts, epithelial cells, and lymphocytes (10). IFN-I therefore links cell-intrinsic danger sensing to inflammatory amplification. By contrast, IFN-γ is the only IFN-II. IFN-γ is produced mainly by activated CD4+ and CD8+ T cells, γδ T cells, natural killer (NK) cells, and natural killer T (NKT) cells (21). Its expression is regulated by cytokines, including IL-12/IL-18 (22), IL-2 (23), IL-15 (24), and IL-27 (25), as well as by transcription factors such as T-bet (26)and Eomes (27). Thus, IFN-I more directly reflects tissue injury-associated danger sensing, whereas IFN-γ more often represents an effector program of activated innate and adaptive immune cells.

IFN-I induction is mainly triggered by activation of pattern recognition receptors (PRRs) by PAMPs or DAMPs. In MI, cardiomyocyte necrosis, mitochondrial dysfunction, nuclear envelope rupture, and cellular stress can release nuclear DNA, RNA, mitochondrial DNA, and other endogenous danger signals (28). These signals are detected by endosomal toll-like receptors (TLR) 3, 7, 8, and 9 (29); the cytosolic RNA sensors retinoic acid-inducible gene I (RIG-I) and melanoma differentiation-associated protein 5 (MDA5) (30); and the DNA sensor cyclic GMP-AMP synthase (cGAS) (31). TLR3 induces IFN expression mainly through the TRIF-TBK1/IKKϵ-IRF3/IRF7 pathway. TLR7, TLR8, and TLR9 depend on the MyD88-IRAK1/4 pathway to activate NF-κB and selected members of the IRF family (32). The SLC15A4-TASL complex provides an important signaling platform for TLR7/8/9-mediated activation of IRF5. After recognition of aberrant RNA, RIG-I and MDA5 interact through their caspase activation and recruitment domains with the mitochondrial outer membrane adaptor mitochondrial antiviral signaling protein (MAVS) (33). This interaction recruits TBK1 and IKKϵ, activates IRF3 and IRF7, and promotes transcription of IFNB1 and selected IFNA genes. After cytosolic DNA is detected, cGAS catalyzes cyclic GMP-AMP (cGAMP) synthesis and activates stimulator of interferon genes (STING), which translocates to Golgi-associated compartments and recruits TBK1 (34). This sequence leads to IRF3 phosphorylation and induction of the IFN-I response. Cell-surface TLR4 can also be activated by DAMPs during sterile tissue injury. Through the MyD88-dependent NF-κB/mitogen-activated protein kinase (MAPK) branch and the TRIF-dependent IRF3 branch, TLR4 links inflammatory signaling to IFN-I induction (35–37). Thus, the IFN-I response after MI is not a simple repetition of an antiviral program but rather a mechanism by which necrotic myocardium and stressed cells convert endogenous injury signals into immune amplification.

After secretion, IFN initiate canonical Janus kinase-signal transducer and activator of transcription (JAK-STAT) signaling through specific receptors on target cells. IFN-I binds the IFNAR1/IFNAR2 receptor complex, activates JAK1 and tyrosine kinase 2 (TYK2), and induces phosphorylation of STAT1 and STAT2 (20). STAT1 and STAT2 then associate with IRF9 to form the interferon-stimulated gene factor 3 (ISGF3) complex, which translocates to the nucleus, binds interferon-stimulated response elements (ISREs), and drives expression of interferon-stimulated genes (ISGs) (38, 39). IFN-γ signals through IFNGR1/IFNGR2, activates JAK1 and JAK2, and induces STAT1 phosphorylation. Phosphorylated STAT1 forms STAT1 homodimers, known as γ-activated factor (GAF), which bind γ-activated sequences (GAS) and initiate target gene transcription (40). Therefore, IFN-I and IFN-γ share the JAK-STAT signaling framework but diverge at the level of transcriptional control. IFN-I primarily uses the ISGF3-ISRE axis, whereas IFN-γ mainly uses the STAT1-GAF-GAS axis. This signaling divergence provides a molecular basis for their different biological effects. The biological effects of IFN signaling depend substantially on the ISGs repertoire induced in a given cell type and disease stage. ISGs are not a single group of antiviral molecules. They include overlapping functional modules: antiviral effectors such as IFITM proteins (41), Mx proteins (42), the OAS-RNase L pathway (43), protein kinase R (PKR), and IFIT proteins (44); signal-sensing and amplification molecules such as RIG-I, MDA5, TLR3, IRF1, IRF7, STAT1, STAT2, and IRF9 (45); and negative regulators that limit the intensity and duration of IFN responses, including suppressor of cytokine signaling (SOCS) proteins, ubiquitin-specific peptidase 18 (USP18), and interferon-stimulated gene 15 (ISG15) (45).

In addition to the canonical JAK-STAT axis, IFN can signal through parallel pathways, including mitogen-activated protein kinase (MAPK), phosphatidylinositol 3-kinase-AKT-mechanistic target of rapamycin (PI3K-AKT-mTOR), and metabolism-related pathways (10). These routes can regulate transcriptional output, protein translation, cell survival, and immunometabolic adaptation. Some ISGs can also be induced in settings of low IFN activity or even in the absence of IFN signaling, through pathways involving IRF, NF-κB, IL-27-STAT1, or TNF-α-IRF1 (46). To limit tissue injury caused by persistent IFN activation, IFN signaling is constrained by several negative-feedback mechanisms, including SOCS proteins (47), protein inhibitor of activated STAT (PIAS) proteins (48), SHP1/2 (49), and the USP18-ISG15 axis (50). This signaling complexity is particularly relevant to postinfarction inflammation. In the setting of cardiomyocyte necrosis, mitochondrial DNA release, and sustained cellular stress, multiple layers of IFN signaling may influence inflammatory cell recruitment, immune-cell reprogramming, fibroblast activation, extracellular matrix deposition, and ventricular remodeling. Therefore, IFN signaling after myocardial infarction should not be viewed simply as an antiviral-like response. It is better understood as a multilayered network that links injury sensing, immune regulation, and tissue repair.

### 2.2 Cross-talk between interferon signaling and inflammation

IFN signaling is not separate from inflammation. It is embedded within PRR mediated innate immune networks and acts together with NF-κB-dependent transcription and inflammasome activation to shape inflammatory outcomes (51). The first level of crosstalk occurs early after PRR activation. TLR, retinoic acid-inducible gene I-like receptor (RLR), and cGAS pathways can activate the TBK1-IRF3/IRF7 axis and the IKK-NF-κB axis in parallel after stimulation by PAMPs or DAMPs (52). The former drives IFN-I transcription, whereas the latter induces inflammatory gene expression. Transcription of the IFNB1 enhancer requires coordinated assembly of IRF, NF-κB, and activator protein 1 (AP-1), indicating that IFN induction and proinflammatory transcription are integrated from the initiation phase (53). A second level of crosstalk arises from IFN-mediated inflammatory priming. IFN-I and IFN-γ induce multiple ISGs and transcriptional regulators through the JAK-STAT pathway, thereby altering the threshold for subsequent PRR responses (54). IFN-γ can remodel enhancer states and chromatin accessibility in macrophages through STAT1 and IRF1, leading to stronger or more sustained transcription of selected TLR-induced NF-κB target genes (55). This amplification is context-dependent and varies according to cell lineage, stimulus type, and disease stage. The inflammasome is a third point of interaction between IFN signaling and inflammation. IFN can enhance infection-related inflammasome responses. In mouse models of gram-negative bacterial infection, IFN-I is required for caspase-11 expression, which supports activation of the noncanonical inflammasome (56). IFN-induced guanylate-binding proteins (GBPs) can promote exposure of pathogen-derived components and inflammasome assembly, thereby increasing infection-associated IL-1β and IL-18 responses (57). The RLR adaptor MAVS can also link IFN signaling to the NLR family pyrin domain containing 3 (NLRP3) inflammasome by promoting mitochondrial localization of NLRP3 and increasing IL-1β production (58). IFN signaling can also restrain excessive inflammasome activation through negative regulatory mechanisms. IFN-I induced pyrin-only protein 3 (POP3) can inhibit activation of absent in melanoma 2 (AIM2)/AIM2-like receptor (ALR) inflammasomes induced by DNA or DNA viruses (59). IFN-β can induce cholesterol 25-hydroxylase, increasing 25-hydroxycholesterol production and limiting inflammasome activation (60). IFN-γ can induce inducible nitric oxide synthase (iNOS) and increase nitric oxide production, which interferes with complete inflammasome assembly (61). IFN-I can also reduce NLRP3 activity through a STAT1-dependent mechanism and decrease caspase-1-mediated maturation of IL-1β (62). Conversely, inflammasomes can regulate IFN pathways. After inflammasome activation, caspase-1 can cleave cGAS and attenuate cGAS-STING -mediated DNA-induced IFN-I production, suggesting that inflammasomes may act as a negative-feedback brake on nucleic acid sensing (63). Thus, IFN-inflammatory crosstalk links nucleic acid sensing, NF-κB-dependent transcription, inflammasome assembly, and tissue inflammatory responses.

## 3 The role of the interferon signaling network in MI

Postinfarction inflammation is not merely a process of injury amplification. It is part of a time-dependent repair program that includes clearance of necrotic tissue, extracellular matrix remodeling, and scar stabilization. This process is generally divided into inflammatory, proliferative, and maturation phases (64). During the inflammatory phase, necrotic cardiomyocytes release large amounts of DAMPs and induce the production of proinflammatory cytokines and chemokines, which recruit neutrophils, monocytes, and macrophages to the infarcted myocardium (65). A controlled inflammatory response is required for clearance of dead cells and matrix debris, whereas excessive inflammation can worsen myocardial injury and promote adverse ventricular remodeling. Repair then shifts toward a proliferative phase characterized by resolution of inflammation, fibroblast activation, and extracellular matrix deposition. This is followed by a maturation phase marked by collagen cross-linking, scar contraction, and stabilization of the fibrotic scar (66). IFN signaling is increasingly viewed as an important regulatory axis linking injury sensing, immune-cell phenotypic remodeling, and structural remodeling of the myocardium (12). After MI, IFN signaling can be induced by DAMPs and by pathways such as cGAS-STING-IRF signaling (67). Its effects vary across immune cells, fibroblasts, and cardiomyocytes. The role of IFN after MI is not uniformly proinflammatory or protective. It depends on IFN subtype, timing of activation, cell type, and the local microenvironment (13). Therefore, examining IFN signaling across the inflammatory, proliferative, and maturation phases of post-MI repair may help clarify the spatial and temporal organization of postinfarction inflammation. It may also provide a rationale for stage-specific and cell-targeted immune modulation after MI (**Figure 2**).

**Figure 2:**
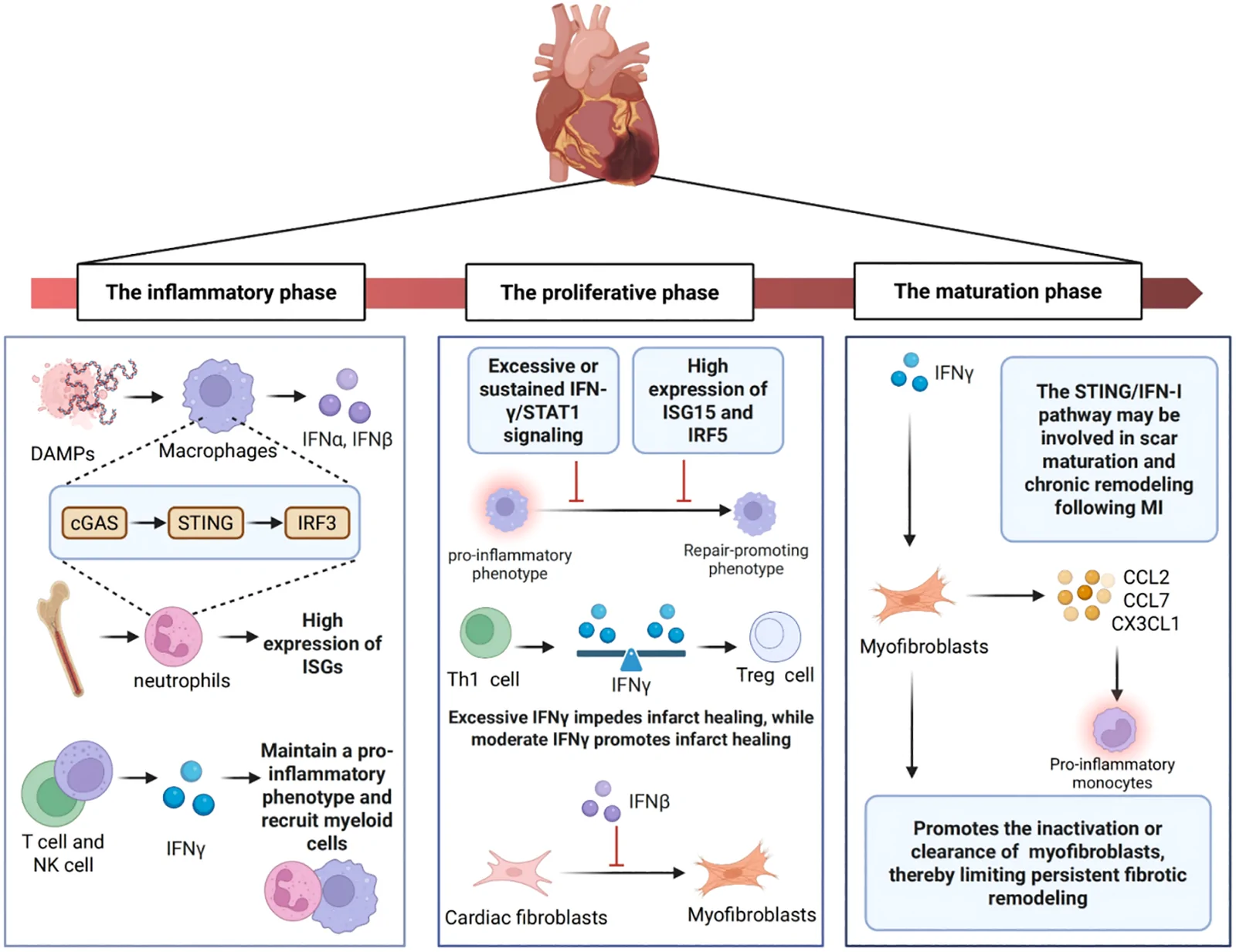
The role of the IFN signaling network at different stages following MI. IFN signaling undergoes dynamic changes during the inflammatory, proliferative, and maturation phases following MI. During the inflammatory phase, necrotic myocardial tissue releases DAMPs and free nucleic acids, which activate the cGAS-STING-IRF3 axis in myeloid cells, inducing the expression of IFN-α/β and ISGs, and synergizing with IFN-γ derived from T cells and NK cells to amplify the recruitment of inflammatory cells. During the proliferative phase, sustained or excessive IFN-γ/STAT1 signaling and high expression of ISG15 and IRF5 can maintain the pro-inflammatory state of macrophages, hindering the transition to a reparative state; conversely, moderate IFN-γ and IFN-β-related signaling may regulate Treg infiltration, resolution of inflammation, and fibroblast activation. In the maturation phase, IFN-γ promotes the inactivation or clearance of myofibroblasts, limiting fibrosis, but may also maintain pro-inflammatory monocyte recruitment by inducing CCL2, CCL7, and CX3CL1; sustained STING/IFN-I/ISG programs may contribute to scar expansion and chronic adverse remodeling. Overall, IFN signaling following MI constitutes an immunoregulatory network linking injury perception, the inflammatory response, fibrosis regulation, and ventricular remodeling.

### 3.1 The inflammatory phase

Clinical studies indicate that the inflammatory phase of MI is accompanied by marked IFN-related immune activation. In patients with acute MI, peripheral blood levels or signatures of IFN-γ, IRF1, IFN-inducible genes, and IFN-related pathways are increased (68–70). Immune repertoire and transcriptomic analyses further suggest tissue-specific T-cell expansion in the ischemic heart. Infiltrating CD8+ T cells may express high levels of IFN-γ, and enrichment of IFN-I responses has been observed in some patients with AMI (69, 71). These clinical findings support an association between IFN signaling and post-MI immune regulation, although most evidence remains observational. Mechanistically, DAMPs released from necrotic cardiomyocytes provide early danger signals that initiate immune activation (72). These include high mobility group box 1 (HMGB1), heat shock proteins (HSPs), extracellular ATP, extracellular matrix degradation products, and cell-free nucleic acids (64). Among these signals, mitochondrial DNA and other forms of cell-free DNA appear closely linked to IFN-I induction in the infarcted myocardium (11, 73). Recent spatial transcriptomic studies also suggest that chromosomal DNA leakage from cardiomyocytes in the border zone is associated with local IFN-I responses (74). These nucleic acid signals can be sensed by macrophages and induce IFN-I and ISG expression through the cGAS-STING pathway (11). A specific population of IFN-inducible cells, composed mainly of monocyte-derived macrophages, has been identified within the infarcted myocardium. After uptake of DNA released from injured cardiomyocytes, these cells activate the cGAS-STING-IRF3 pathway and mount an IFN-I response, thereby promoting immune-cell recruitment and amplifying local inflammatory cascades (11). Accordingly, inhibition of STING and its downstream TBK1-IRF3 axis in macrophages reduces inflammatory injury after MI and improves subsequent ventricular dilation, myocardial fibrosis, and systolic dysfunction in experimental models (75). Activation of cGAS-STING signaling can also promote a shift of macrophages toward an M1-like proinflammatory phenotype, whereas pathway inhibition favors an M2-like reparative phenotype and attenuates pathological cardiac remodeling (73). Whether this phenotypic shift is directly mediated by downstream IFN signaling remains uncertain and requires further study. Beyond the local cardiac response, MI can initiate a myeloid IFN program in the bone marrow. This process gives neutrophils and monocytes a high-ISG phenotype before they enter the infarcted heart (76). Further studies suggest that the cGAS-STING pathway in neutrophils may act upstream of this bone marrow-derived IFN program (77). cGAS-STING signaling not only promotes expansion of early neutrophil progenitors and precursor neutrophils but also increases ISG expression in neutrophils, placing them in a primed state before cardiac infiltration. In line with this model, inhibition of neutrophil cGAS-STING reduces the risk of cardiac rupture and death after MI, suggesting a role in amplifying acute inflammatory injury (77).

Unlike IFN-I, which is mainly driven by DNA sensing during the inflammatory phase, IFN-γ reflects crosstalk between lymphoid and myeloid cells. After MI, CD4+ T cells, CD8+ T cells, and NK cells in the heart can produce IFN-γ (78). IFN-γ enhances inflammation through metabolic reprogramming of macrophages and suppresses macrophage repair-associated gene expression by inhibiting the transcription factor MAF, thereby helping maintain a proinflammatory macrophage phenotype (79, 80). IFN-γ can also drive monocyte differentiation toward proinflammatory macrophages (81). Under selected stimuli, macrophages may produce IFN-γ themselves, creating an autocrine amplification loop (82). IFN-γ released from the infarcted heart also acts on LysM+ myeloid cells to sustain neutrophil recruitment and neutrophil-mediated chemokine networks, which support the sequential infiltration of inflammatory and reparative monocytes (83). In post-MI macrophages, the E3 ubiquitin ligase RNF149 limits IFN-γ signaling by promoting ubiquitination and degradation of IFNGR1 (84). Loss of RNF149 enhances infiltration of proinflammatory monocytes and macrophages, accelerates the decline of reparative subsets, and worsens cardiomyocyte apoptosis and infarct healing (84). Taken together, excessive IFN-mediated inflammation during the inflammatory phase of MI appears to be a multilayered immune program initiated by nucleic acid DAMP sensing, mediated in part by cGAS-STING, and amplified through both IFN-I and IFN-γ pathways. The cGAS-STING axis mainly links damaged nucleic acid sensing to IFN-I-dependent myeloid activation, whereas IFN-γ further amplifies local inflammation through lymphoid-myeloid crosstalk. More selective regulation of the cGAS-STING-IFN-I axis and the IFN-γ-myeloid cell axis may help limit excessive inflammatory injury after MI while preserving repair, but this concept still requires validation in clinically relevant settings.

### 3.2 The proliferative phase

The proliferative phase marks the transition from acute inflammation to inflammation resolution and scar formation after MI (85). During this phase, macrophages, lymphocytes, fibroblasts, and vascular cells participate in clearance of necrotic tissue, extracellular matrix deposition, and vascular remodeling (86). These processes help maintain structural stability of the infarcted region and reduce the risk of cardiac rupture. In this context, IFN signaling cannot be classified simply as injurious or reparative. Clinical studies have reported that higher early levels of IFN-α, IFN-β, and IFN-γ after MI are associated with subsequent adverse ventricular remodeling (87, 88). In contrast, other data suggest that IFN-I-related transcriptional signature in circulating monocytes from patients with MI is associated with more favorable left ventricular remodeling (89). These findings suggest that increased systemic IFN levels and cell-specific IFN responses may have different biological and clinical implications. During inflammation resolution, macrophages are important effector cells that link removal of necrotic tissue to tissue repair. Experimental studies have shown that exogenous IFN-α increases the proportion of inflammatory monocytes after MI in rats and impairs cardiac healing (89). Sustained or excessive IFN-γ/STAT1 signaling can maintain a proinflammatory macrophage program and delay transition toward a reparative state (90). The prostaglandin D2-DP1 axis inhibits IFN-γ receptor mediated JAK2/STAT1 activation, limits proinflammatory polarization, and promotes inflammation resolution (91).

These findings suggest that IFN-γ signaling during repair requires controlled negative regulation rather than persistent activation. At the same time, IFN-related pathways may contribute to inflammation resolution under selected conditions. In non-MI models of resolving inflammation, IFN-β induces macrophages with high phagocytic activity and enhances efferocytosis by upregulating CD36 (92). Whether this mechanism operates in the local myocardial microenvironment during the proliferative phase after MI remains to be established. At the downstream effector level, timely reduction of IFN-related molecules may be required for inflammation resolution. Single-cell studies have identified ISG15-high M1-like macrophages in infarcted myocardium, where they help maintain a proinflammatory state; silencing ISG15 promotes cardiac repair (93). Other studies have shown that IRF5 is highly expressed in early post-MI macrophages and supports a proinflammatory phenotype. Silencing macrophage IRF5 promotes inflammation resolution and improves infarct healing (94). These findings suggest that IFN-related downstream nodes, including ISG15 and IRF5, may contribute to persistence of inflammation during the proliferative phase. In addition to macrophages, CD4+ T cells regulate inflammation resolution and tissue repair after MI in a subset-, source-, and time-dependent manner (95). On the one hand, T helper 1 cells and their IFN-γ signaling can impair infarct healing by suppressing myofibroblast activation (96). On the other hand, regulatory T cells (Tregs) limit excessive inflammation, regulate monocyte/macrophage differentiation, and promote infarct healing (97). Local, controlled administration of IFN-γ can increase Treg infiltration in injured myocardium and reduce fibrosis, thereby limiting maladaptive remodeling (98). Thus, the issue during the proliferative phase is not whether IFN signaling should be broadly inhibited or enhanced, but how its activity is maintained within an appropriate range in specific cells and at specific time points.

Fibroblast activation and expansion are central features of cardiac repair during the proliferative phase after MI. In the healing infarct, activated cardiac fibroblasts and myofibroblasts are major sources of structural collagen, and reparative fibrosis supports scar formation (99). Transforming growth factor β (TGF-β) signaling is an important driver of myofibroblast differentiation and extracellular matrix synthesis (100). Within this setting, IFN signaling may regulate fibroblast activation. In cultured cardiac fibroblasts stimulated with TGF-β1, IFN-β reduces α-smooth muscle actin (α-SMA) expression, type I collagen synthesis, cell migration, and collagen gel contraction through STAT1/STAT2 signaling (101). These findings suggest a potential antifibrotic effect of IFN-β through inhibition of myofibroblast differentiation and matrix contraction. IFN-β may also regulate fibroblast plasticity. Activation of RNA-sensing receptors can induce IFN-β production in fibroblasts and promote their reprogramming toward cardiomyocyte-like cells (102). However, in the *in vivo* MI environment, macrophage-derived IFN-β activates STAT1 phosphorylation in fibroblasts through IFNAR. Phosphorylated STAT1 interacts with the reprogramming factor GATA4 and reduces its chromatin occupancy at cardiac gene regulatory regions, thereby inhibiting fibroblast-to-cardiomyocyte conversion (103). These findings indicate that IFN-I affects cardiac fibroblast activation and plasticity during the proliferative phase, but whether this effect promotes or limits healing remains uncertain. The outcome probably depends on cell type, signal source, timing, and signaling intensity. Overall, IFN signaling influences inflammation resolution, scar formation, and ventricular remodeling during the proliferative phase after MI. Available evidence supports a bidirectional role. IFN responses may help resolve inflammation, regulate Treg infiltration, and limit excessive myofibroblast activation, thereby supporting organized scar formation. Conversely, IFN signaling may maintain proinflammatory macrophage programs, inhibit myofibroblast differentiation, or suppress cardiomyocyte-like reprogramming, delaying tissue repair. The role of IFN signaling in proliferative-phase cardiac repair is therefore best understood as a dynamic balance among IFN subtype, source cell, target cell, timing, and signaling intensity, allowing inflammation resolution and stable scar formation to proceed in sequence.

### 3.3 The maturation phase

The maturation phase marks the transition from extracellular matrix deposition to scar stabilization, vascular maturation, and termination of tissue remodeling after MI. At this stage, the main process is no longer further amplification of myofibroblast activity. Rather, repair requires preservation of the necessary matrix scaffold while myofibroblasts exit a highly proliferative, high α-smooth muscle actin (α-SMA), matrix-producing state and acquire a matrifibrocyte-like phenotype characterized by lower proliferation, reduced α-SMA expression, and matrix maintenance (104). In this phase, IFN signaling may help regulate termination of the myofibroblast response. *In vitro* studies show that IFN-γ inhibits TGF-β1 induced fibroblast-to-myofibroblast differentiation and reduces the expression of collagen I/III, α-SMA, and matrix cross-linking molecules, including LOX-1 and LOXL-2. These findings suggest that IFN-γ may limit excessive fibrosis (105). Other studies have shown that IFN-γ induces STAT1 activation and indoleamine 2,3-dioxygenase (IDO) expression in human cardiac myofibroblasts. Through tryptophan depletion, this response causes G0/G1 cell-cycle arrest, suppresses cell proliferation, and reduces α-SMA expression. When IDO activity is inhibited, IFN-γ can further induce myofibroblast apoptosis through the IRF1/Fas/Fas ligand axis (106). These data suggest that IFN-γ may promote myofibroblast inactivation or clearance during scar maturation, thereby limiting persistent fibrotic remodeling. IFN-γ may also affect late repair by altering the immunochemotactic function of fibroblasts. In the presence of TGF-β1, IFN-γ suppresses the fibrotic phenotype of fibroblasts but increases secretion of CCL2, CCL7, and CX3CL1 and reduces matrix metalloproteinase 2 (MMP-2) expression and activity (105). Conditioned medium from these fibroblasts preferentially recruits proinflammatory monocytes rather than reparative monocytes (105). This finding suggests that IFN-γ can shift fibroblasts from matrix-producing cells toward inflammation-promoting cells. Although this may restrict matrix deposition, it may also sustain recruitment of proinflammatory monocytes, delay inflammatory shutdown, and interfere with scar maturation. If this imbalance persists into chronic ischemic heart failure, sustained IFN-γ related adaptive immune responses may further contribute to adverse remodeling. In mouse models of chronic ischemic cardiomyopathy after MI, Tregs expand and acquire an IFN-γ+ proinflammatory T helper 1 like phenotype, with loss of immunosuppressive function and increased antiangiogenic and profibrotic activity. Depletion of these dysfunctional Tregs reduces left ventricular dilation, cardiac hypertrophy, and interstitial fibrosis and improves neovascularization (107). Clinical studies have also identified tissue-specific, clonally expanded T-cell populations in the hearts of patients with ischemic heart failure, characterized mainly by T helper 1 enrichment, increased CD8+ T cells, and high expression of IFN-γ, granzyme B, and perforin (69). These findings suggest that IFN-γ signaling may have antifibrotic effects during scar maturation, but persistent IFN-γ activity may also link low-grade inflammation, fibrotic expansion, and progression of ischemic heart failure. In addition to IFN-γ, STING/IFN-I related pathways may participate in scar maturation and chronic remodeling after MI. Pharmacologic inhibition of STING at 3 weeks after MI improves left ventricular systolic function, reduces cardiac hypertrophy, collagen deposition, and scar expansion, and downregulates interferon-stimulated genes such as Ifi44 and Cxcl10 (108). These findings suggest that the STING-dependent IFN/ISG program is not only a marker of acute inflammation but may continue to contribute to fibrotic remodeling in the infarcted region and adverse ventricular remodeling. Therefore, intervention in STING-related signaling during the maturation phase may represent a potential approach to limiting scar expansion and delaying progression to ischemic heart failure. Overall, IFN signaling appears to influence termination of the repair response and stabilization of the infarct scar during the maturation phase after MI.

## 4 The therapeutic potential and limitations of interferon

To date, several recombinant IFNs have been used in the treatment of malignant tumors, infectious diseases, multiple sclerosis, and immunodeficiency-related disorders, but no IFN-based drug has been approved for MI (109). Different IFN subtypes have distinct clinical contexts. Recombinant IFN-α2b has been used as adjuvant therapy for high-risk melanoma (110). Recombinant IFN-β1b is one of the disease-modifying therapies for multiple sclerosis (111). IFN-γ1b is approved mainly for reducing the risk of severe infections in chronic granulomatous disease and delaying disease progression in severe malignant osteopetrosis (112). Safety data from previous IFN therapies indicate that exogenous IFN exposure may be associated with cardiac toxic effects. Reported adverse events include arrhythmia, MI, reduced left ventricular systolic function, cardiomyopathy, pericarditis, and pericardial effusion (113). These events have mostly been reported in settings of long-term systemic treatment for cancer or viral hepatitis, often with confounding from infection, systemic inflammation, and concomitant medications. Thus, the potential cardiovascular risks of exogenous IFNs should not be dismissed, but toxicity associated with prolonged systemic exposure cannot be directly extrapolated to short-course, local, or phase-specific interventions for MI. Preclinical data suggest that IFN-γ may have context-dependent protective effects. In a porcine model of cardiopulmonary bypass associated myocardial ischemia-reperfusion injury, a single dose of IFN-γ administered 24 hours before ischemia improved early left ventricular function after reperfusion, reduced leakage of cardiac troponin I and creatine kinase-MB, and decreased cardiomyocyte apoptosis (114). This finding suggests that IFN-γ preconditioning may attenuate ischemic myocardial injury. However, this evidence comes from a predictable ischemia preconditioning model and cannot be directly extended to clinical treatment after acute MI. The translational value of IFNs in MI should therefore be framed as a narrow therapeutic-window problem rather than as long-term systemic immunomodulation. Future studies need to define IFN subtype, dose, delivery route, and timing, and then determine whether short exposure around ischemia or reperfusion can induce protective immune reprogramming without causing systemic inflammatory amplification or cardiotoxicity. Targeting the downstream JAK-STAT pathway provides another approach to limiting pathological IFN signaling. Preclinical studies have shown that the JAK2 inhibitor AG490 downregulates TNF-α and NF-κB, reduces infarct size, and attenuates inflammation and adverse ventricular remodeling (115). The JAK1/2 inhibitor ruxolitinib has also shown potential cardioprotective effects, but direct clinical evidence in patients with MI remains limited (116). In addition, ruxolitinib has been associated with nonsignificant trends toward increased MI, hypertension, and ischemic stroke (117, 118). This inconsistency partly explains why currently available JAK inhibitors cannot be directly translated into treatment for ischemic heart disease.

Given that both exogenous IFN intervention and downstream JAK-STAT inhibition are limited by issues of specificity and safety, and considering the bidirectional effects of IFN signaling during the proliferative and maturation phases, the cGAS-STING axis which has a clearly pro-inflammatory role during the inflammatory phase holds greater translational potential. The cGAS-STING axis is an important pathway for cytosolic DNA sensing and IFN-I induction. Targeting this axis may limit pathological IFN-I signaling and inflammatory amplification at the level of damage DNA sensing, without broadly blocking downstream JAK-STAT pathways that also support tissue repair and basal immune homeostasis (119). In MI, this strategy is supported by relatively consistent preclinical evidence. The STING inhibitor H-151 limits infarct expansion and scar formation, improves left ventricular systolic function, and reduces cardiac hypertrophy and collagen deposition in transient left anterior descending coronary artery occlusion models (120). RU.521 and scutellarin inhibit cGAS-STING activation and downregulate apoptosis-related molecules under hypoxia/reoxygenation or ischemia/reperfusion conditions (121). Other interventions, including the endoplasmic reticulum stress inhibitor 4-phenylbutyric acid (4-PBA) (122), tanshinone IIA combined with astragaloside IV (123), panaxatriol saponin (75), and curcumol (124), have also been reported to reduce ischemic injury after MI through inhibition of STING signaling. However, cGAS-STING may have different roles in cardiomyocytes, macrophages, endothelial cells, and fibroblasts. In cardiomyocytes and fibroblasts, it may contribute to remodeling by regulating apoptosis, senescence, autophagy, and fibrosis (125). Clinical translation of cGAS-STING targeted therapy should therefore avoid prolonged systemic inhibition and instead focus on short-course treatment, tissue-targeted delivery, and disease-stage selection.

Overall, exogenous IFN administration and broad inhibition of downstream hubs such as JAK-STAT are constrained by pathway pleiotropy. These approaches may suppress inflammation but also impair myocardial ischemic tolerance and repair. By contrast, targeting the upstream cGAS-STING axis may more selectively intercept injury-driven immune overactivation and may offer a more practical route for myocardial protection and improved remodeling. Development of tissue-specific STING inhibitors using nanoparticle-based delivery, restricted to the acute phase of MI, may help maximize cardiac protection while reducing adverse effects (**Figure 3**).

**Figure 3:**
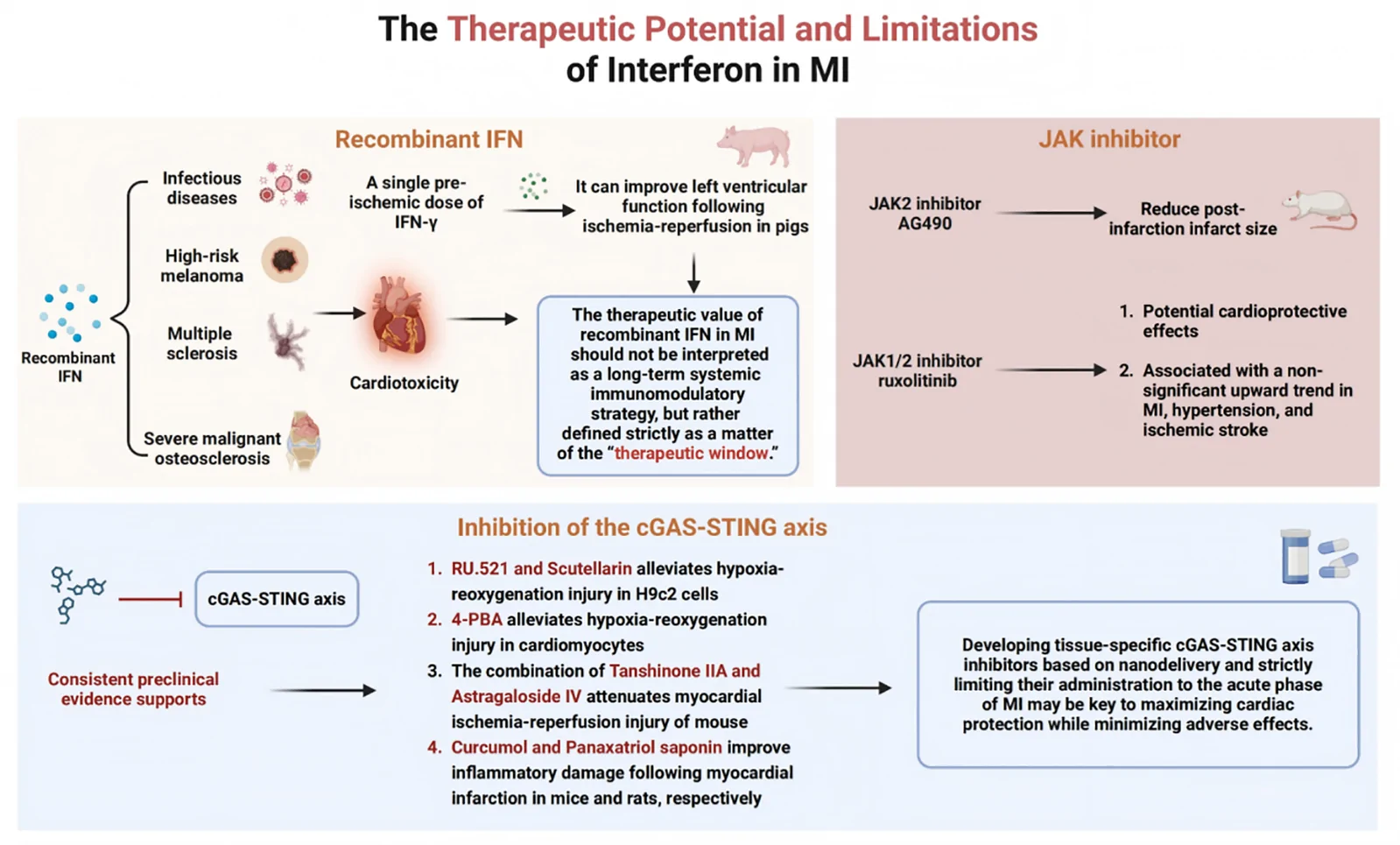
The translational potential and limitations of IFN intervention in MI. Various recombinant IFNs have been used to treat infectious diseases, high-risk melanoma, multiple sclerosis, and severe malignant osteosclerosis, among other conditions. Previous long-term, systemic IFN therapy has suggested a potential for cardiotoxicity; however, preclinical studies have shown that a single pre-ischemic administration of IFN-γ can improve early left ventricular function following ischemia/reperfusion in pigs. Therefore, the use of exogenous IFN in MI should be limited to a specific “therapeutic window.” Targeting the downstream JAK-STAT axis also presents a paradox of potential cardioprotective effects and cardiac injury. In contrast, interventions targeting the cGAS-STING axis in preclinical studies have consistently demonstrated cardioprotective effects. Interventions such as H-151, RU.521, 4-PBA, baicalin, tanshinone IIA combined with astragaloside IV, curcumin, and panaxatriol saponin can alleviate ischemic injury, inflammatory amplification, and fibrotic remodeling by inhibiting cGAS-STING signaling. Tissue-specific cGAS-STING axis inhibitors delivered via nanotechnology may represent a key strategy for providing cardioprotective effects with a low risk of immunosuppression.

## 5 Limitations and future prospects

Although increasing evidence suggests that IFN and related signaling pathways participate in post-MI inflammation, immune-cell remodeling, fibrosis regulation, and ventricular remodeling, the field remains largely at the stage of mechanistic study and preclinical validation. Several barriers still limit clinical translation. IFN signaling is not a simple linear cascade but a complex, multistep process encompassing injury sensing, signal transduction, ISG output, and negative-feedback regulation, with its biological effects after MI depending on IFN subtype, cellular source, disease stage, signaling intensity, and duration. Future studies should therefore move beyond the broad question of whether IFN signaling is beneficial or harmful. A more useful approach is to define how IFN signaling affects myocardial injury, inflammation resolution, scar formation, and ventricular remodeling within specific pathological stages and tissue contexts after MI. Current evidence on IFN-mediated regulation of myocardial injury and repair is still derived mainly from animal models and *in vitro* models of simulated ischemia. Longitudinal data from patients remain limited. Large prospective clinical cohorts are needed to assess IFN-related signatures in peripheral blood mononuclear cells, plasma cytokine profiles, and available cardiac tissue. These studies may clarify whether IFN signaling can serve as a biomarker for early risk identification, prognostic stratification, or prediction of treatment response. The post-MI cardiac microenvironment is highly heterogeneous, and different cell types may show similar, complementary, or opposing IFN-response programs. Future work should integrate single-cell transcriptomics, spatial multiomics, and *in vivo* lineage tracing to define IFN production, IFN responsiveness, and downstream effector programs across cell populations and disease stages. Such studies should also map the spatial distribution of IFN-active cells and their patterns of intercellular communication within the infarcted myocardium. The bidirectional nature of IFN signaling remains one of the main challenges for clinical translation. Therapeutic strategies targeting IFN-related pathways should not rely on long-term, systemic, or nonspecific inhibition. Instead, they will require clear selection of the treatment window and pathological state. In the early phase after acute MI, short-course inhibition of aberrant nucleic acid sensing and pathological IFN amplification may reduce excessive inflammation and myocardial injury. The goal is not simply to enhance or suppress IFN signaling, but to identify and selectively modulate pathogenic IFN programs while preserving its physiological roles in debris clearance, immune homeostasis, and coordination of repair. Drug development targeting the cGAS-STING pathway may offer a way to limit pathological IFN signaling while better preserving systemic immune homeostasis and cardiac repair capacity. Overall, IFN signaling provides a useful basis for understanding post-MI inflammation, fibrosis progression, and ventricular remodeling, but its translation into therapy remains uncertain. Future studies should focus on precise, short-course, and reversible modulation of pathogenic IFN signaling according to disease stage, cell type, and local microenvironment, rather than broad activation or inhibition of the entire pathway.

## References

[1] Martin SS Aday AW Allen NB Almarzooq ZI Anderson CAM Arora P . 2025 heart disease and stroke statistics: A report of US and global data from the American Heart Association. Circulation. (2025) 151:e41–e660. doi: 10.1161/CIR.0000000000001303 39866113 PMC12256702

[2] Matter MA Paneni F Libby P Frantz S Stähli BE Templin C . Inflammation in acute myocardial infarction: The good, the bad and the ugly. Eur Heart J. (2024) 45:89–103. doi: 10.1093/eurheartj/ehad486 37587550 PMC10771378

[3] Nahrendorf M Swirski FK Aikawa E Stangenberg L Wurdinger T Figueiredo J-L . The healing myocardium sequentially mobilizes two monocyte subsets with divergent and complementary functions. J Exp Med. (2007) 204:3037–47. doi: 10.1084/jem.20070885 18025128 PMC2118517

[4] Panizzi P Swirski FK Figueiredo JL Waterman P Sosnovik DE Aikawa E . Impaired infarct healing in atherosclerotic mice with Ly-6C(hi) monocytosis. J Am Coll Cardiol. (2010) 55:1629–38. doi: 10.1016/j.jacc.2009.08.089 20378083 PMC2852892

[5] Ridker PM Everett BM Thuren T MacFadyen JG Chang WH Ballantyne C . Antiinflammatory therapy with canakinumab for atherosclerotic disease. N Engl J Med. (2017) 377:1119–31. doi: 10.1056/NEJMoa1707914 28845751

[6] Tardif J-C Kouz S Waters DD Bertrand OF Diaz R Maggioni AP . Efficacy and safety of low-dose colchicine after myocardial infarction. N Engl J Med. (2019) 381:2497–505. doi: 10.1056/NEJMoa1912388 31733140

[7] Jolly SS d’Entremont M-A Lee SF Mian R Tyrwhitt J Kedev S . Colchicine in acute myocardial infarction. N Engl J Med. (2025) 392:633–42. doi: 10.1056/NEJMoa2405922 39555823

[8] Abbate A Wohlford GF Del Buono MG Chiabrando JG Markley R Turlington J . Interleukin-1 blockade with anakinra and heart failure following ST-segment elevation myocardial infarction: Results from a pooled analysis of the VCUART clinical trials. Eur Heart J Cardiovasc Pharmacother. (2022) 8:503–10. doi: 10.1093/ehjcvp/pvab075 34617567 PMC9366639

[9] Sadler AJ Williams BRG . Interferon-inducible antiviral effectors. Nat Rev Immunol. (2008) 8:559–68. doi: 10.1038/nri2314 18575461 PMC2522268

[10] Wen C Lan Q Wang Y Ni Y Wong AST Liu D . Interferon signaling pathways in health and disease. Mol BioMed. (2025) 6:135. doi: 10.1186/s43556-025-00381-5 41359111 PMC12686292

[11] King KR Aguirre AD Ye Y-X Sun Y Roh JD Ng RP . IRF3 and type I interferons fuel a fatal response to myocardial infarction. Nat Med. (2017) 23:1481–7. doi: 10.1038/nm.4428 29106401 PMC6477926

[12] Tran DT Batchu SN Advani A . Interferons and interferon-related pathways in heart disease. Front Cardiovasc Med. (2024) 11:1357343. doi: 10.3389/fcvm.2024.1357343 38665231 PMC11043610

[13] Crow MK Ronnblom L . Type I interferons in host defence and inflammatory diseases. Lupus Sci Med. (2019) 6:e000336. doi: 10.1136/lupus-2019-000336 31205729 PMC6541752

[14] Isaacs A Lindenmann J . Pillars article: Virus interference. I. The interferon. Proc R Soc Lond B Biol Sci. 1957. 147: 258-267. J Immunol. (2015) 195:1911–20. doi: 10.1093/jimmunol/195.5.1911 26297790

[15] Lai L Zhang A Yang B Charles EJ Kron IL Yang Z . Plasmacytoid dendritic cells mediate myocardial ischemia/reperfusion injury by secreting type I interferons. J Am Heart Assoc. (2021) 10:e020754. doi: 10.1161/JAHA.121.020754 34325534 PMC8475660

[16] Cantaert T Baeten D Tak PP van Baarsen LGM . Type I IFN and TNFα cross-regulation in immune-mediated inflammatory disease: Basic concepts and clinical relevance. Arthritis Res Ther. (2010) 12:219. doi: 10.1186/ar3150 21062511 PMC2991015

[17] Zanin N Viaris de Lesegno C Lamaze C Blouin CM . Interferon receptor trafficking and signaling: Journey to the cross roads. Front Immunol. (2020) 11:615603. doi: 10.3389/fimmu.2020.615603 33552080 PMC7855707

[18] Boehmer D Zanoni I . Interferons in health and disease. Cell. (2025) 188:4480–504. doi: 10.1016/j.cell.2025.06.044 40845809 PMC12380125

[19] Wittling MC Cahalan SR Levenson EA Rabin RL . Shared and unique features of human interferon-beta and interferon-alpha subtypes. Front Immunol. (2020) 11:605673. doi: 10.3389/fimmu.2020.605673 33542718 PMC7850986

[20] Shemesh M Lochte S Piehler J Schreiber G . IFNAR1 and IFNAR2 play distinct roles in initiating type I interferon-induced JAK-STAT signaling and activating STATs. Sci Signal. (2021) 14:eabe4627. doi: 10.1126/scisignal.abe4627 34813358

[21] Burke JD Young HA . IFN-γ: A cytokine at the right time, is in the right place. Semin Immunol. (2019) 43:101280. doi: 10.1016/j.smim.2019.05.002 31221552 PMC7367502

[22] Rackov G Tavakoli Zaniani P Colomo Del Pino S Shokri R Monserrat J Alvarez-Mon M . Mitochondrial reactive oxygen is critical for IL-12/IL-18-induced IFN-γ production by CD4+ T cells and is regulated by Fas/FasL signaling. Cell Death Dis. (2022) 13:531. doi: 10.1038/s41419-022-04907-5 35668079 PMC9170726

[23] Van Gelder RD Gokhale NS Genoyer E Omelia DS Anderson SK Young HA . Interleukin-2-mediated NF-κB-dependent mRNA splicing modulates interferon gamma protein production. EMBO Rep. (2025) 26:16–35. doi: 10.1038/s44319-024-00324-1 39578552 PMC11724048

[24] Cimpean M Keppel MP Gainullina A Fan C Sohn H Schedler NC . IL-15 priming alters IFN-γ regulation in murine NK cells. J Immunol. (2023) 211:1481–93. doi: 10.4049/jimmunol.2300283 37747317 PMC10873103

[25] Wiesheu R Edwards SC Hedley A Hall H Tosolini M Fares da Silva MGF . IL-27 maintains cytotoxic Ly6C+ γδ T cells that arise from immature precursors. EMBO J. (2024) 43:2878–907. doi: 10.1038/s44318-024-00133-1 38816652 PMC11251046

[26] Wong P Foltz JA Chang L Neal CC Yao T Cubitt CC . T-BET and EOMES sustain mature human NK cell identity and antitumor function. J Clin Invest. (2023) 133:e162530. doi: 10.1172/JCI162530 37279078 PMC10313375

[27] Xu Z Ma W Wang J Chen H Li H Yin Z . Nuclear HMGB1 is critical for CD8 T cell IFN-γ production and anti-tumor immunity. Cell Rep. (2024) 43:114591. doi: 10.1016/j.celrep.2024.114591 39116204

[28] Shah M Yellon DM Davidson SM . The role of extracellular DNA and histones in ischaemia-reperfusion injury of the myocardium. Cardiovasc Drugs Ther. (2020) 34:123–31. doi: 10.1007/s10557-020-06946-6 32062794 PMC7093382

[29] Miyake K Shibata T Fukui R Sato R Saitoh S-I Murakami Y . Nucleic acid sensing by Toll-like receptors in the endosomal compartment. Front Immunol. (2022) 13:941931. doi: 10.3389/fimmu.2022.941931 35812450 PMC9259784

[30] Onomoto K Onoguchi K Yoneyama M . Regulation of RIG-I-like receptor-mediated signaling: Interaction between host and viral factors. Cell Mol Immunol. (2021) 18:539–55. doi: 10.1038/s41423-020-00602-7 33462384 PMC7812568

[31] Xu Y Chen C Liao Z Xu P . cGAS-STING signaling in cell death: Mechanisms of action and implications in pathologies. Eur J Immunol. (2023) 53:e2350386. doi: 10.1002/eji.202350386 37424054

[32] Kawai T Sato S Ishii KJ Coban C Hemmi H Yamamoto M . Interferon-alpha induction through Toll-like receptors involves a direct interaction of IRF7 with MyD88 and TRAF6. Nat Immunol. (2004) 5:1061–8. doi: 10.1038/ni1118 15361868

[33] Rehwinkel J Gack MU . RIG-I-like receptors: Their regulation and roles in RNA sensing. Nat Rev Immunol. (2020) 20:537–51. doi: 10.1038/s41577-020-0288-3 32203325 PMC7094958

[34] Pan J Fei C-J Hu Y Wu X-Y Nie L Chen J . Current understanding of the cGAS-STING signaling pathway: Structure, regulatory mechanisms, and related diseases. Zool Res. (2023) 44:183–218. doi: 10.24272/j.issn.2095-8137.2022.464 36579404 PMC9841179

[35] Kagan JC Su T Horng T Chow A Akira S Medzhitov R . TRAM couples endocytosis of Toll-like receptor 4 to the induction of interferon-beta. Nat Immunol. (2008) 9:361–8. doi: 10.1038/ni1569 18297073 PMC4112825

[36] Schultz TE Mathmann CD Domínguez Cadena LC Muusse TW Kim H Wells JW . TLR4 endocytosis and endosomal TLR4 signaling are distinct and independent outcomes of TLR4 activation. EMBO Rep. (2025) 26:2740–66. doi: 10.1038/s44319-025-00444-2 40204912 PMC12116916

[37] Stilp A-C Scherer M König P Fürstberger A Kestler HA Stamminger T . The chromatin remodeling protein ATRX positively regulates IRF3-dependent type I interferon production and interferon-induced gene expression. PloS Pathog. (2022) 18:e1010748. doi: 10.1371/journal.ppat.1010748 35939517 PMC9387936

[38] Nowicka H Sekrecka A Blaszczyk K Kluzek K Chang C-Y Wesoly J . ISGF3 and STAT2/IRF9 control basal and IFN-induced transcription through genome-wide binding of phosphorylated and unphosphorylated complexes to common ISRE-containing ISGs. Int J Mol Sci. (2023) 24:17635. doi: 10.3390/ijms242417635 38139463 PMC10743977

[39] Schiefer S Hale BG . Proximal protein landscapes of the type I interferon signaling cascade reveal negative regulation by PJA2. Nat Commun. (2024) 15:4484. doi: 10.1038/s41467-024-48800-5 38802340 PMC11130243

[40] Han J Wu M Liu Z . Dysregulation in IFN-γ signaling and response: The barricade to tumor immunotherapy. Front Immunol. (2023) 14:1190333. doi: 10.3389/fimmu.2023.1190333 37275859 PMC10233742

[41] Majdoul S Compton AA . Lessons in self-defence: Inhibition of virus entry by intrinsic immunity. Nat Rev Immunol. (2022) 22:339–52. doi: 10.1038/s41577-021-00626-8 34646033 PMC8511856

[42] Betancor G . You shall not pass: MX2 proteins are versatile viral inhibitors. Vaccines (Basel). (2023) 11:930. doi: 10.3390/vaccines11050930 37243034 PMC10224399

[43] Harioudh MK Perez J Chong Z Nair S So L McCormick KD . Oligoadenylate synthetase 1 displays dual antiviral mechanisms in driving translational shutdown and protecting interferon production. Immunity. (2024) 57:446–461.e7. doi: 10.1016/j.immuni.2024.02.002 38423012 PMC10939734

[44] Smart A Gilmer O Caliskan N . Translation inhibition mediated by interferon-stimulated genes during viral infections. Viruses. (2024) 16:1097. doi: 10.3390/v16071097 39066259 PMC11281336

[45] Schneider WM Chevillotte MD Rice CM . Interferon-stimulated genes: A complex web of host defenses. Annu Rev Immunol. (2014) 32:513–45. doi: 10.1146/annurev-immunol-032713-120231 24555472 PMC4313732

[46] Swaraj S Tripathi S . Interference without interferon: Interferon-independent induction of interferon-stimulated genes and its role in cellular innate immunity. mBio. (2024) 15:e0258224. doi: 10.1128/mbio.02582-24 39302126 PMC11481898

[47] Croker BA Krebs DL Zhang J-G Wormald S Willson TA Stanley EG . SOCS3 negatively regulates IL-6 signaling *in vivo*. Nat Immunol. (2003) 4:540–5. doi: 10.1038/ni931 12754505

[48] Magalhaes J Tresse E Ejlerskov P Hu E Liu Y Marin A . PIAS2-mediated blockade of IFN-β signaling: A basis for sporadic Parkinson disease dementia. Mol Psychiatry. (2021) 26:6083–99. doi: 10.1038/s41380-021-01207-w 34234281 PMC8758491

[49] An H Zhao W Hou J Zhang Y Xie Y Zheng Y . SHP-2 phosphatase negatively regulates the TRIF adaptor protein-dependent type I interferon and proinflammatory cytokine production. Immunity. (2006) 25:919–28. doi: 10.1016/j.immuni.2006.10.014 17157040

[50] Vasou A Nightingale K Cetkovská V Scheler J Bamford CGG Andrejeva J . ISG15-dependent stabilisation of USP18 is necessary but not sufficient to regulate type I interferon signalling in humans. Eur J Immunol. (2025) 55:e202451651. doi: 10.1002/eji.202451651 39931755 PMC11811815

[51] Kopitar-Jerala N . The role of interferons in inflammation and inflammasome activation. Front Immunol. (2017) 8:873. doi: 10.3389/fimmu.2017.00873 28791024 PMC5525294

[52] Chen R Zou J Chen J Zhong X Kang R Tang D . Pattern recognition receptors: function, regulation and therapeutic potential. Signal Transduct Target Ther. (2025) 10:216. doi: 10.1038/s41392-025-02264-1 40640149 PMC12246121

[53] Iwanaszko M Kimmel M . NF-κB and IRF pathways: cross-regulation on target genes promoter level. BMC Genomics. (2015) 16:307. doi: 10.1186/s12864-015-1511-7 25888367 PMC4430024

[54] Barrat FJ Crow MK Ivashkiv LB . Interferon target-gene expression and epigenomic signatures in health and disease. Nat Immunol. (2019) 20:1574–83. doi: 10.1038/s41590-019-0466-2 31745335 PMC7024546

[55] Ivashkiv LB . IFNγ: signalling, epigenetics and roles in immunity, metabolism, disease and cancer immunotherapy. Nat Rev Immunol. (2018) 18:545–58. doi: 10.1038/s41577-018-0029-z 29921905 PMC6340644

[56] Rathinam VAK Vanaja SK Waggoner L Sokolovska A Becker C Stuart LM . TRIF licenses caspase-11-dependent NLRP3 inflammasome activation by gram-negative bacteria. Cell. (2012) 150:606–19. doi: 10.1016/j.cell.2012.07.007 22819539 PMC3660860

[57] Pilla DM Hagar JA Haldar AK Mason AK Degrandi D Pfeffer K . Guanylate binding proteins promote caspase-11-dependent pyroptosis in response to cytoplasmic LPS. Proc Natl Acad Sci USA. (2014) 111:6046–51. doi: 10.1073/pnas.1321700111 24715728 PMC4000848

[58] Subramanian N Natarajan K Clatworthy MR Wang Z Germain RN . The adaptor MAVS promotes NLRP3 mitochondrial localization and inflammasome activation. Cell. (2013) 153:348–61. doi: 10.1016/j.cell.2013.02.054 23582325 PMC3632354

[59] Khare S Ratsimandresy RA de Almeida L Cuda CM Rellick SL Misharin AV . The PYRIN domain-only protein POP3 inhibits ALR inflammasomes and regulates responses to infection with DNA viruses. Nat Immunol. (2014) 15:343–53. doi: 10.1038/ni.2829 24531343 PMC4123781

[60] Reboldi A Dang EV McDonald JG Liang G Russell DW Cyster JG . Inflammation. 25-hydroxycholesterol suppresses interleukin-1-driven inflammation downstream of type I interferon. Science. (2014) 345:679–84. doi: 10.1126/science.1254790 25104388 PMC4289637

[61] Mishra BB Rathinam VAK Martens GW Martinot AJ Kornfeld H Fitzgerald KA . Nitric oxide controls the immunopathology of tuberculosis by inhibiting NLRP3 inflammasome-dependent processing of IL-1β. Nat Immunol. (2013) 14:52–60. doi: 10.1038/ni.2474 23160153 PMC3721324

[62] Guarda G Braun M Staehli F Tardivel A Mattmann C Förster I . Type I interferon inhibits interleukin-1 production and inflammasome activation. Immunity. (2011) 34:213–23. doi: 10.1016/j.immuni.2011.02.006 21349431

[63] Wang Y Ning X Gao P Wu S Sha M Lv M . Inflammasome activation triggers caspase-1-mediated cleavage of cGAS to regulate responses to DNA virus infection. Immunity. (2017) 46:393–404. doi: 10.1016/j.immuni.2017.02.011 28314590

[64] Hilgendorf I Frantz S Frangogiannis NG . Repair of the infarcted heart: Cellular effectors, molecular mechanisms and therapeutic opportunities. Circ Res. (2024) 134:1718–51. doi: 10.1161/CIRCRESAHA.124.323658 38843294 PMC11164543

[65] Chalise U Becirovic-Agic M Lindsey ML . The cardiac wound healing response to myocardial infarction. WIREs Mech Dis. (2023) 15:e1584. doi: 10.1002/wsbm.1584 36634913 PMC10077990

[66] Toba H Halade GV DeLeon-Pennell KY Chiao YA Yabluchanskiy A Holm Nielsen S . Cellular and molecular signals of cardiac wound healing after myocardial infarction. Am J Physiol Heart Circ Physiol. (2026) 330:H594–609. doi: 10.1152/ajpheart.00891.2025 41533392 PMC12914773

[67] Lei C Tan Y Ni D Peng J Yi G . cGAS-STING signaling in ischemic diseases. Clin Chim Acta. (2022) 531:177–82. doi: 10.1016/j.cca.2022.04.003 35398249

[68] Yan W Song Y Zhou L Jiang J Yang F Duan Q . Immune cell repertoire and their mediators in patients with acute myocardial infarction or stable angina pectoris. Int J Med Sci. (2017) 14:181–90. doi: 10.7150/ijms.17119 28260995 PMC5332848

[69] Tang T-T Zhu Y-C Dong N-G Zhang S Cai J Zhang L-X . Pathologic T-cell response in ischaemic failing hearts elucidated by T-cell receptor sequencing and phenotypic characterization. Eur Heart J. (2019) 40:3924–33. doi: 10.1093/eurheartj/ehz516 31365073

[70] van Blokland IV Oelen R Groot HE Benjamins JW Pekayvaz K Losert C . Single-cell dissection of the immune response after acute myocardial infarction. Circ Genom Precis Med. (2024) 17:e004374. doi: 10.1161/CIRCGEN.123.004374 38752343 PMC11188632

[71] Piacentini L Myasoedova VA Chiesa M Vavassori C Moschetta D Valerio V . Whole-blood transcriptome unveils altered immune response in acute myocardial infarction patients with aortic valve sclerosis. Arterioscler Thromb Vasc Biol. (2024) 44:452–64. doi: 10.1161/ATVBAHA.123.320106 38126173 PMC10805353

[72] Silvis MJM Kaffka Genaamd Dengler SE Odille CA Mishra M van der Kaaij NP Doevendans PA . Damage-associated molecular patterns in myocardial infarction and heart transplantation: The road to translational success. Front Immunol. (2020) 11:599511. doi: 10.3389/fimmu.2020.599511 33363540 PMC7752942

[73] Cao DJ Schiattarella GG Villalobos E Jiang N May HI Li T . Cytosolic DNA sensing promotes macrophage transformation and governs myocardial ischemic injury. Circulation. (2018) 137:2613–34. doi: 10.1161/CIRCULATIONAHA.117.031046 29437120 PMC5997506

[74] Ninh VK Calcagno DM Yu JD Zhang B Taghdiri N Sehgal R . Spatially clustered type I interferon responses at injury borderzones. Nature. (2024) 633:174–81. doi: 10.1038/s41586-024-07806-1 39198639 PMC11374671

[75] Yao H He Q Wei S Xiang L Luo Y Huang C . Targeted inhibition of macrophage STING signaling alleviates inflammatory injury and ventricular remodeling in acute myocardial infarction. Acta Pharm Sin B. (2025) 15:4030–46. doi: 10.1016/j.apsb.2025.06.014 40893684 PMC12399207

[76] Calcagno DM Ng RP Toomu A Zhang C Huang K Aguirre AD . The myeloid type I interferon response to myocardial infarction begins in bone marrow and is regulated by Nrf2-activated macrophages. Sci Immunol. (2020) 5:eaaz1974. doi: 10.1126/sciimmunol.aaz1974 32978242 PMC7808338

[77] Zhu J Ruan X Mangione MC Parra P Chen G Su X . The cGAS-STING pathway promotes acute ischemia-induced neutropoiesis and neutrophil priming in the bone marrow. Basic Res Cardiol. (2025) 120:677–705. doi: 10.1007/s00395-025-01111-2 40332608 PMC12976895

[78] Kologrivova I Shtatolkina M Suslova T Ryabov V . Cells of the immune system in cardiac remodeling: Main players in resolution of inflammation and repair after myocardial infarction. Front Immunol. (2021) 12:664457. doi: 10.3389/fimmu.2021.664457 33868315 PMC8050340

[79] Wang F Zhang S Jeon R Vuckovic I Jiang X Lerman A . Interferon gamma induces reversible metabolic reprogramming of M1 macrophages to sustain cell viability and pro-inflammatory activity. EBioMedicine. (2018) 30:303–16. doi: 10.1016/j.ebiom.2018.02.009 29463472 PMC5953001

[80] Kang K Park SH Chen J Qiao Y Giannopoulou E Berg K . Interferon-γ represses M2 gene expression in human macrophages by disassembling enhancers bound by the transcription factor MAF. Immunity. (2017) 47:235–250.e4. doi: 10.1016/j.immuni.2017.07.017 28813657 PMC5568089

[81] Luque-Martin R Angell DC Kalxdorf M Bernard S Thompson W Eberl HC . IFN-γ drives human monocyte differentiation into highly proinflammatory macrophages that resemble a phenotype relevant to psoriasis. J Immunol. (2021) 207:555–68. doi: 10.4049/jimmunol.2001310 34233910

[82] Darwich L Coma G Peña R Bellido R Blanco EJJ Este JA . Secretion of interferon-gamma by human macrophages demonstrated at the single-cell level after costimulation with interleukin (IL)-12 plus IL-18. Immunology. (2009) 126:386–93. doi: 10.1111/j.1365-2567.2008.02905.x 18759749 PMC2669819

[83] Finger S Knorr M Molitor M Schüler R Garlapati V Waisman A . A sequential interferon gamma directed chemotactic cellular immune response determines survival and cardiac function post-myocardial infarction. Cardiovasc Res. (2019) 115:1907–17. doi: 10.1093/cvr/cvz092 30949687

[84] Huang C-K Chen Z Zhou Z Chen S Chen L Li L . RNF149 destabilizes IFNGR1 in macrophages to favor postinfarction cardiac repair. Circ Res. (2024) 135:518–36. doi: 10.1161/CIRCRESAHA.123.324023 38989590

[85] Halade GV Lee DH . Inflammation and resolution signaling in cardiac repair and heart failure. EBioMedicine. (2022) 79:103992. doi: 10.1016/j.ebiom.2022.103992 35405389 PMC9014358

[86] Prabhu SD Frangogiannis NG . The biological basis for cardiac repair after myocardial infarction: From inflammation to fibrosis. Circ Res. (2016) 119:91–112. doi: 10.1161/CIRCRESAHA.116.303577 27340270 PMC4922528

[87] Eyyupkoca F Eyerci N Ozkan C Kocak A Gok M Ercan K . The role of interferons in cardiac remodeling after acute myocardial infarction. J Coll Physicians Surg Pak. (2022) 32:837–42. doi: 10.29271/jcpsp.2022.07.837 35795928

[88] Altintas MS Eyerci N Sivri S Felekoglu MA Karayigit O Demirtas B . The role of temporal changes of pro-inflammatory cytokines in the development of adverse cardiac remodeling after ST-elevation myocardial infarction. Postepy Kardiol Interwencyjnej. (2022) 18:217–27. doi: 10.5114/aic.2022.120938 36751290 PMC9885240

[89] Ter Horst EN Krijnen PAJ Hakimzadeh N Robbers LFHJ Hirsch A Nijveldt R . Elevated monocyte-specific type I interferon signalling correlates positively with cardiac healing in myocardial infarct patients but interferon alpha application deteriorates myocardial healing in rats. Basic Res Cardiol. (2018) 114:1. doi: 10.1007/s00395-018-0709-7 30443679 PMC6244641

[90] Xu S Li H Han J Xu Y Li N Che W . Klf9 promotes the repair of myocardial infarction by regulating macrophage recruitment and polarization. JCI Insight. (2025) 10:e187072. doi: 10.1172/jci.insight.187072 40198141 PMC12128982

[91] Kong D Shen Y Liu G Zuo S Ji Y Lu A . PKA regulatory IIα subunit is essential for PGD2-mediated resolution of inflammation. J Exp Med. (2016) 213:2209–26. doi: 10.1084/jem.20160459 27621415 PMC5030806

[92] Zeituni-Timor O Soboh S Zaid A Yaseen H Kumaran Satyanarayanan S Abu Zeid M . IFN-β and ARTS deficiency promote the generation of hyper-efferocytic Ly6C+ macrophages in resolving inflammation in male mice. Commun Biol. (2025) 8:1269. doi: 10.1038/s42003-025-08707-3 40849518 PMC12375040

[93] Shen J Fan Z Ren L Cheng Y Wu C . Interferon-stimulated gene 15 small interfering RNA-loaded polarized mesoporous silica nanocarriers remodel the immune microenvironment to ameliorate post-myocardial infarction heart failure. Mater Today Bio. (2026) 36:102631. doi: 10.1016/j.mtbio.2025.102631 41560785 PMC12813121

[94] Courties G Heidt T Sebas M Iwamoto Y Jeon D Truelove J . *In vivo* silencing of the transcription factor IRF5 reprograms the macrophage phenotype and improves infarct healing. J Am Coll Cardiol. (2014) 63:1556–66. doi: 10.1016/j.jacc.2013.11.023 24361318 PMC3992176

[95] Hofmann U Beyersdorf N Weirather J Podolskaya A Bauersachs J Ertl G . Activation of CD4+ T lymphocytes improves wound healing and survival after experimental myocardial infarction in mice. Circulation. (2012) 125:1652–63. doi: 10.1161/CIRCULATIONAHA.111.044164 22388323

[96] Yan X Zhang H Fan Q Hu J Tao R Chen Q . Dectin-2 deficiency modulates Th1 differentiation and improves wound healing after myocardial infarction. Circ Res. (2017) 120:1116–29. doi: 10.1161/CIRCRESAHA.116.310260 28193608

[97] Gladow N Hollmann C Weirather J Ding X Burkard M Uehlein S . Role of CD4+ T-cells for regulating splenic myelopoiesis and monocyte differentiation after experimental myocardial infarction. Basic Res Cardiol. (2024) 119:261–75. doi: 10.1007/s00395-024-01035-3 38436707 PMC11008073

[98] Ramjee V Li D Manderfield LJ Liu F Engleka KA Aghajanian H . Epicardial YAP/TAZ orchestrate an immunosuppressive response following myocardial infarction. J Clin Invest. (2017) 127:899–911. doi: 10.1172/JCI88759 28165342 PMC5330722

[99] Venugopal H Hanna A Humeres C Frangogiannis NG . Properties and functions of fibroblasts and myofibroblasts in myocardial infarction. Cells. (2022) 11:1386. doi: 10.3390/cells11091386 35563692 PMC9102016

[100] Frangogiannis NG . Transforming growth factor-β in myocardial disease. Nat Rev Cardiol. (2022) 19:435–55. doi: 10.1038/s41569-021-00646-w 34983937

[101] Bolivar S Espitia-Corredor JA Olivares-Silva F Valenzuela P Humeres C Anfossi R . In cardiac fibroblasts, interferon-beta attenuates differentiation, collagen synthesis, and TGF-β1-induced collagen gel contraction. Cytokine. (2021) 138:155359. doi: 10.1016/j.cyto.2020.155359 33160814

[102] Wang X Baksh SS Pratt RE Dzau VJ Hodgkinson CP . Modifying miRs for effective reprogramming of fibroblasts to cardiomyocytes. Mol Ther Nucleic Acids. (2024) 35:102160. doi: 10.1016/j.omtn.2024.102160 38495845 PMC10943962

[103] Wang H Yang J Cai Y Zhao Y . Macrophages suppress cardiac reprogramming of fibroblasts *in vivo* via IFN-mediated intercellular self-stimulating circuit. Protein Cell. (2024) 15:906–29. doi: 10.1093/procel/pwae013 38530808 PMC11637486

[104] Fu X Khalil H Kanisicak O Boyer JG Vagnozzi RJ Maliken BD . Specialized fibroblast differentiated states underlie scar formation in the infarcted mouse heart. J Clin Invest. (2018) 128:2127–43. doi: 10.1172/JCI98215 29664017 PMC5957472

[105] Pappritz K Savvatis K Koschel A Miteva K Tschöpe C Van Linthout S . Cardiac (myo)fibroblasts modulate the migration of monocyte subsets. Sci Rep. (2018) 8:5575. doi: 10.1038/s41598-018-23881-7 29615815 PMC5882911

[106] Lee J-W Oh JE Rhee K-J Yoo B-S Eom YW Park SW . Co-treatment with interferon-γ and 1-methyl tryptophan ameliorates cardiac fibrosis through cardiac myofibroblasts apoptosis. Mol Cell Biochem. (2019) 458:197–205. doi: 10.1007/s11010-019-03542-7 31006829 PMC6616223

[107] Bansal SS Ismahil MA Goel M Zhou G Rokosh G Hamid T . Dysfunctional and proinflammatory regulatory T-lymphocytes are essential for adverse cardiac remodeling in ischemic cardiomyopathy. Circulation. (2019) 139:206–21. doi: 10.1161/CIRCULATIONAHA.118.036065 30586716 PMC6322956

[108] Rech L Abdellatif M Pöttler M Stangl V Mabotuwana N Hardy S . Small molecule STING inhibition improves myocardial infarction remodeling. Life Sci. (2022) 291:120263. doi: 10.1016/j.lfs.2021.120263 34971697

[109] George PM Badiger R Alazawi W Foster GR Mitchell JA . Pharmacology and therapeutic potential of interferons. Pharmacol Ther. (2012) 135:44–53. doi: 10.1016/j.pharmthera.2012.03.006 22484806

[110] Ives NJ Suciu S Eggermont AMM Kirkwood J Lorigan P Markovic SN . Adjuvant interferon-α for the treatment of high-risk melanoma: An individual patient data meta-analysis. Eur J Cancer. (2017) 82:171–83. doi: 10.1016/j.ejca.2017.06.006 28692949

[111] Jakimovski D Kolb C Ramanathan M Zivadinov R Weinstock-Guttman B . Interferon β for multiple sclerosis. Cold Spring Harb Perspect Med. (2018) 8:a032003. doi: 10.1101/cshperspect.a032003 29311124 PMC6211378

[112] Razaghi A Owens L Heimann K . Review of the recombinant human interferon gamma as an immunotherapeutic: Impacts of production platforms and glycosylation. J Biotechnol. (2016) 240:48–60. doi: 10.1016/j.jbiotec.2016.10.022 27794496

[113] Sonnenblick M Rosin A . Cardiotoxicity of interferon. A review of 44 cases. Chest. (1991) 99:557–61. doi: 10.1378/chest.99.3.557 1704826

[114] Yan X Qiu W Jia B Zhong H Li X Chen Z . Myocardial protection by interferon-γ late preconditioning during cardiopulmonary bypass-associated myocardial ischemia-reperfusion in pigs. Oncol Rep. (2013) 30:2145–52. doi: 10.3892/or.2013.2707 24002640

[115] Zhang S Liu X Goldstein S Li Y Ge J He B . Role of the JAK/STAT signaling pathway in the pathogenesis of acute myocardial infarction in rats and its effect on NF-κB expression. Mol Med Rep. (2013) 7:93–8. doi: 10.3892/mmr.2012.1159 23128561

[116] Reyes Gaido OE Pavlaki N Granger JM Mesubi OO Liu B Lin BL . An improved reporter identifies ruxolitinib as a potent and cardioprotective CaMKII inhibitor. Sci Transl Med. (2023) 15:eabq7839. doi: 10.1126/scitranslmed.abq7839 37343080 PMC11022683

[117] Dunn R Long E Gagnon LL Harrison C Yang Y O’Sullivan J . Treatment of myeloproliferative neoplasms with Janus kinase inhibitors: A meta-analysis of cardiovascular safety. EJHaem. (2025) 6:e70000. doi: 10.1002/jha2.70000 39944793 PMC11815325

[118] Verstovsek S Mesa RA Gotlib J Gupta V DiPersio JF Catalano JV . Long-term treatment with ruxolitinib for patients with myelofibrosis: 5-year update from the randomized, double-blind, placebo-controlled, phase 3 COMFORT-I trial. J Hematol Oncol. (2017) 10:55. doi: 10.1186/s13045-017-0417-z 28228106 PMC5322633

[119] Hopfner K-P Hornung V . Molecular mechanisms and cellular functions of cGAS-STING signalling. Nat Rev Mol Cell Biol. (2020) 21:501–21. doi: 10.1038/s41580-020-0244-x 32424334

[120] Hu S Gao Y Gao R Wang Y Qu Y Yang J . The selective STING inhibitor H-151 preserves myocardial function and ameliorates cardiac fibrosis in murine myocardial infarction. Int Immunopharmacol. (2022) 107:108658. doi: 10.1016/j.intimp.2022.108658 35278833

[121] Li J-K Song Z-P Hou X-Z . Scutellarin ameliorates ischemia/reperfusion injury-induced cardiomyocyte apoptosis and cardiac dysfunction via inhibition of the cGAS-STING pathway. Exp Ther Med. (2023) 25:155. doi: 10.3892/etm.2023.11854 36911381 PMC9996299

[122] Li Y Lin H Tang H Zhu K Zhou Z Zeng Z . The STING-IRF3 signaling pathway, mediated by endoplasmic reticulum stress, contributes to impaired myocardial autophagic flux after ischemia/reperfusion. J Cardiovasc Pharmacol. (2023) 82:389–99. doi: 10.1097/FJC.0000000000001465 37851150

[123] Zhai P Chen Q Wang X Ouyang X Yang M Dong Y . The combination of Tanshinone IIA and Astragaloside IV attenuates myocardial ischemia-reperfusion injury by inhibiting the STING pathway. Chin Med. (2024) 19:34. doi: 10.1186/s13020-024-00908-y 38419127 PMC10900662

[124] Yang N Yu G Liu T Dang Y Deng P Lei Z . Direct inhibition of macrophage sting signaling by curcumol protects against myocardial infarction via attenuating the inflammatory response. Phytomedicine. (2025) 138:156403. doi: 10.1016/j.phymed.2025.156403 39889491

[125] Feng L Zhang F Cao J Xiong W . Role of cGAS-STING pathway in fibrotic disease. Pharmacol Res. (2025) 221:107971. doi: 10.1016/j.phrs.2025.107971 41005587

